# SARS-CoV-2 Omicron strain exhibits potent capabilities for immune evasion and viral entrance

**DOI:** 10.1038/s41392-021-00852-5

**Published:** 2021-12-17

**Authors:** Xiantao Zhang, Shijian Wu, Bolin Wu, Qirui Yang, Achun Chen, Yuzhuang Li, Yiwen Zhang, Ting Pan, Hui Zhang, Xin He

**Affiliations:** 1 Institute of Human Virology, Key Laboratory of Tropical Disease Control of Ministry Education, Guangdong Engineering Research Center for Antimicrobial Agent and Immunotechnology, The Eighth Affiliated Hospital, Zhongshan School of Medicine, Sun Yat-sen University, Guangzhou, Guangdong 510080 China; 2 Center for Infection and Immunity Study, School of Medicine, Sun Yat-sen University, Shenzhen, Guangdong 518107 China; 3 National Guangzhou Laboratory, Bio-Island, Guangzhou, Guangdong 510320 China

**Keywords:** Infection, Infectious diseases

**Dear Editor**,

Severe acute respiratory syndrome coronavirus 2 (SARS-CoV-2) Omicron strain (as known as B.1.1.529) was identified in Botswana and South Africa in early November 2021 and was later defined as a new variant of concern (VOC) by the World Health Organization on November 26, 2021.^1,2^ To date, the confirmed cases of Omicron have been reported in more than 38 countries, and the number of cases appears to be rapidly increasing. SARS-CoV-2 Omicron could give rise to the fourth wave of the COVID-19 epidemic spreading around the world, following the D614G, Beta/Gamma, and Delta VOCs. Here, we showed Omicron variant causes serious immune escape from the convalescent sera from COVID-19 patients, still needs angiotensin-converting enzyme 2 (ACE2) as its receptor and exhibits a significantly increased infectivity. These substantial lines of evidence should attract broad attention to monitor its epidemic and accelerate the development of Omicron-targeted vaccines and therapeutic antibodies.

Omicron is the most heavily mutated variant to emerge so far.^3^ These mutations may result in large conformation alterations, which could affect transmissibility, disease severity, and capacity for immune evasion. Although Omicron seems to exhibit a tendency of high transmissibility, which has already caused some panic in the whole world but lacks substantial virological or immunological evidence. Herein, we took advantage of our mature SARS-CoV-2 research platform and performed the neutralizing assays with Omicron Spike (S)-packaged pseudotyped viruses to evaluate whether the SARS-CoV-2 Omicron variant could escape the cross-neutralization of convalescent sera from the patients infected with the early strains or the Delta strain. To this end, we constructed an Omicron S protein-expressing plasmid (GISAID: EPI_ISL_6752027) for packaging the pseudotyped SARS-CoV-2 S/HIV-1 viruses, as well as that with various S proteins derived from B.1.1.7 (Alpha), B.1.351 (Beta), and B.1.617.2 (Delta), which were constructed previously.^4^ The luciferase gene was incorporated into the HIV-1 vector and can be expressed after infection with the pseudotyped viruses. We incubated various pseudotyped SARS-CoV-2 S/HIV-1 viruses with the convalescent sera from 27 early strain (D614 or G614)-infected patients, or 18 Delta strain-infected patients, followed by the detection of infectivity. The “seesaw effect” between the early strain and Delta strain in the neutralizing titer of respective convalescent sera was confirmed. Surprisingly, both convalescent sera from early strain- or Delta-infected patients demonstrated quite low neutralization capacity against pseudotyped Omicron viruses. The neutralizing antibody titer in the convalescent sera from early strain-infected patients for Omicron variant decreased 36 times, while that in the convalescent sera from Delta strain-infected patients decreased 39 times, which were significantly lower than the neutralizing antibody titers for other variants (Fig. 1a, b). These results indicate a significant immune escape from an existed protection established by virus infection or vaccination. The vigorous immune escape of the Omicron variant was also in consistent with the reports that the initial confirmed cases have already got fully vaccinated, or infected with SARS-CoV-2 previously.

**Fig. 1:**
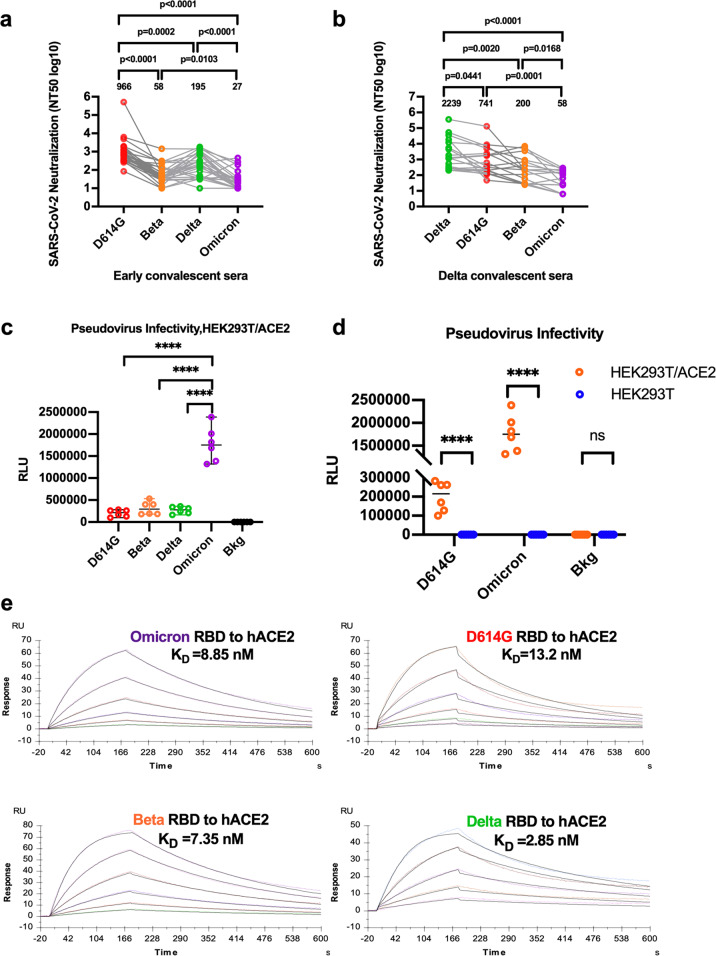
Omicron variant exhibits potent capabilities for immune evasion and viral entrance. **a** The neutralizing antibodies (nAbs) titers of early convalescent sera against SARS-CoV-2 pseudoviruses (D614G/Beta/Delta/Omicron) were determined and represented as half-maximal inhibitory concentrations (NT50). (*n* = 27, Adjusted *p* values were calculated by one-way ANOVA with Tukey’s multiple comparisons test *p < 0.05, ***p < 0.001). **b** NT50 of each Delta convalescent sera against each pseudotyped virus (*n* = 18 Adjusted *p* values were calculated by one-way ANOVA with Tukey’s multiple comparisons test *p < 0.05, ***p < 0.001). **c**, **d** The Omicron pseudoviruses were more infectious than other pseudotyped viruses in HEK293T/ACE2 cells. The relative luminescence unit (RLU) of Luc reporter gene expression was standardized as the p24 content of the pseudoviruses (the p24 content of the pseudovirus in the supernatants: D614G = 287 ng/mL, Beta = 306 ng/mL, Delta = 282 ng/ml, Omicron = 289 ng/ml). The background (Bkg) RLU was measured in the wells that received the cells but not the pseudoviruses. **e** Surface plasmon resonance (SPR) was used to analyze the affinity of Omicron_RBD, D614G_RBD, Beta_RBD, and Delta_RBD bound to ACE2. The K_D_ values were calculated by the software BIAevaluation and shown was a mean of three independent experiments

To further identify the influence of mutants on the S protein of Omicron variant to the potential virus infectivity, we infected the HEK293T cells stably expressing the ACE2 receptor and quantified the titer of various pseudotyped SARS-CoV-2 S/HIV-1 viruses. Compared to Beta or Delta, there was an approximately tenfold increase in the virus infectivity from Omicron S protein-mediated viral entrance (Fig. 1c). We then found that the Omicron S/HIV-1 pseudotyped viruses cannot enter HEK293T cell without ACE2, indicating that Omicron still used ACE2 as its major receptor although Omicron contains 15 mutations in the receptor-binding domain (RBD) region (residues Arg319–Phe541) (Fig. 1d). Further, because the entrance of SARS-CoV-2 to the target cells relies on the binding of RBD to ACE2, we applied the Surface Plasmon Resonance (SPR) assay to investigate whether the binding of RBD to ACE2 was influenced by the high mutations in the RBD region of the Omicron variant. The RBDs of Omicron, Beta, Delta, and D614 were constructed with 6 × His-tagged at their C termini, followed by expression and purification from HEK293-F cells to keep the glycosylation modifications which were essential to bind to ACE2 to mediate host-cell entry. Compared to the RBDs of Beta or Delta variants, the binding of RBD of the Omicron variant to ACE2 exhibited a similar binding affinity (Fig. 1e). Collectively, compared with Beta or Delta S-bearing viruses, the entrance of Omicron S-bearing viruses into HEK293T-ACE2 cells was indeed enhanced by the highly mutated Spike. However, as the binding affinity of the RBD region to ACE2 is not significantly increased, this increased capability could be due to the mutation(s) in other areas of S protein.

Given the high mutations in the RBD region, it is worrisome for a possible “receptor shift” that ACE2 may no longer be the receptor for Omicron. In this report, we found that the infection of Omicron still needs ACE2 expression. The binding affinity of RBD and ACE2 maintains the nanomolar level which is similar among Beta, Delta, and Omicron. Because of the strict dependence of ACE2 for RBD, it seems that all the variants have already reached the nanomolar scale and are difficult for the virus to further improve. Compared with three major epidemic variants, Beta, Delta, or D614G, Omicron variant carries more than thirty amino acid mutations in the S protein. Surprisingly, almost half are located in RBD, including the shared K417N, N501Y with Beta, the shared T478K with Delta, as well as the D614G in almost all the VOCs, where T478K has already caused breakthrough infection in vaccinated people.^5^ Moreover, mutation of E484A occurs in Omicron while the E484K in Beta, which contributed to the evasion of antibody neutralization. The basic mutation in poly-basic cleavage site (PBCS), P681H, has been demonstrated to benefit Furin-mediated cleavage in S protein, which could potentially enhance the infectivity.^4^ Additional new mutations, including G339D, S371L, S373P, S375F, N440K, G446S, S477N, Q493K, G496S, Q498R, and Y505H, occurs in the RBD of the Omicron variant. Further, the receptor-binding motif (RBM, 438aa–508aa) contains 12 mutations in the Omicron strain, half of which was located around the N501 at the C-terminus. These mutants could lead to large conformation changes which increase the capabilities for immune evasion. Regarding the detailed structural analysis for Omicron RBD and ACE2 interaction, more structural biology work is required for future studies.

In this report, we first demonstrated that the newly emerging SARS-CoV-2 Omicron variant significantly escaped from the neutralization of convalescent sera from early strain- or Delta- infected patients, indicating that these recovered patients were still vulnerable to the newly emerging variant strain. Consistently, many initial confirmed Omicron cases were already fully vaccinated or previously infected, revealing the occurrence of breakthrough reinfection and the immune escape of Omicron variant.^6^ It has been well known that neutralizing antibody plays a key role in anti-SARS-CoV-2 immunity. The major targets of neutralizing antibodies against SARS-CoV-2 locates in S protein, especially in the RBD region. The high immune evasion we observed for Omicron is probably associated with the high mutated S protein and RBD region. As ACE2 is still required for its infectivity, the RBD-targeted vaccine or therapeutics is still effective, although adjustment is necessary. As its entrance capability is significantly increased, its transmissibility could also increase, albeit more epidemic evidence is required to verify this possibility. Taken together, our results demonstrate that the Omicron variant severely threatens the current prophylaxis and therapeutic strategy. Because the Omicron variant may have already spread over for 1 or 2 months, the whole world should continue closely and carefully to monitor its epidemic, particularly in the virus mutation evolution and the real transmissibility rate. Importantly, the Omicron-targeted vaccine and drug development are urgently needed to overcome this newly-merged VOC of SARS-CoV-2.

## Supplementary information

SUPPLEMENTAL MATERIAL AND METHOD

## Data Availability

The data are available from the corresponding author on reasonable request.
