# Supplementary material for: SARS-CoV-2 Omicron strain exhibits potent capabilities for immune evasion and viral entrance
Source: Signal Transduct Target Ther. 2021 Dec 17;6:430. doi: 10.1038/s41392-021-00852-5 (PMC8678971; doi:10.1038/s41392-021-00852-5)
Supplement: Supplementary file 1 — SUPPLEMENTAL MATERIAL AND METHOD [file 41392_2021_852_MOESM1_ESM.docx]

Supplementary Materials for

SARS-CoV-2 Omicron strain exhibits potent capabilities for immune evasion and viral entrance

Xiantao Zhang^1^, Shijian Wu^1^, Bolin Wu^1^, Qirui Yang^1^, Achun Chen^1^, Yuzhuang Li^1^, Yiwen Zhang^1^, Ting Pan^1,2^, Hui Zhang^1,3,^ * and Xin He^1,^ *

Correspondence to: Xin He; E-mail: [hexin59@mail.sysu.edu.cn](mailto:hexin59@mail.sysu.edu.cn)

Hui Zhang; E-mail: [zhangh92@mail.sysu.edu.cn](mailto:zhangh92@mail.sysu.edu.cn)

**This PDF file includes:**

Materials and Methods

Materials and Methods

**Ethics statements**

The Ethics Review Boards of Sun Yat-sen University, Guangzhou 8th People’s Hospital and Fifth Affiliated Hospital of Sun Yat-sen University approved this study. Convalescent sera of COVID-19 patients were obtained from Guangzhou 8th People’s Hospital and Fifth Affiliated Hospital of Sun Yat-sen University. All the convalescent sera were positive for RBD-specific antibodies^1,2^. All the participants were given written informed consent with approval of the Ethics Committees.

**PCR-based mutagenesis**

To generate SARS-CoV-2 pseudovirus, the spike protein-expressing plasmids were constructed. The gene encoding the Spike protein of B.1.1.529 virus (GISAID: EPI_ISL_6752027) was codon-optimized and assembled by multi-round overlapping polymerase chain reaction (PCR), followed by cloning into pcDNA3.1 vector. The expressing plasmids for D614G mutation, B.1.351(GISAID: EPI_ISL_678597), or B.1.1.617.2 (GISAID: EPI_ISL_2131531) variants were previously constructed by us^1,2^. All the Spike protein-expressing constructs were verified by Sanger sequencing.

**Pseudotyped virus neutralization assay**

The assay was performed based upon our previous procedures^1,2^. Briefly, HEK293T cells were co-transfected with the psPAX2 (Addgene) plasmid, the lentiviral plasmid expressing luciferase (Addgene), and the plasmid expressing the respective mutant spike proteins by using polyethyleneimine (PEI, Sigma). Forty-eight hours after transfection, the culture supernatant was collected and filtered with a 0.20 µm filter, and then stored at -80°C. Virus titration was performed by serially diluting the viral infection of hACE2-293T cells, and the infectivity was measured by detecting luminescence. The sera of all immunized animals were serially diluted and incubated with a pre-titrated amount of pseudotyped SARS-CoV-2 virus at 37 °C for 1 h. Subsequently, the serum/virus mixture was added to the wells containing 2×10^4^ hACE2-293T cells and incubated at 37°C in 5% CO2 for 48 h. Then the cells were lysed with lysis buffer (Promega), and the lysate was used to detect the relative luminescence unit (RLU) in the photometer (Promega) to measure the luciferase activity. GraphPad Prism 8.0 software was used to analyze the serum neutralizing antibody titers of the pseudotyped viruses.

**Pseudotyped virus infectivity**

HIV-1 p24 content (produced by the backbone vectors) was quantified using the Alliance p24 ELISA Kit (PerkinElmer Health Sciences, Cat# NEK050B001KT). The relative luminescence unit (RLU) of Luc reporter gene expression is standardized as the p24 content of the pseudovirus (the p24 content of the pseudovirus in supernatants: G614 = 287 ng/mL, B.1.351 = 306 ng/mL, B.1.617 .2 = 282 ng/ml, B.1.1.529=289 ng/ml). Sixty ul of each pseudovirus were added to the wells containing 2×10^4^ hACE2-293T cells and incubated at 37 °C in 5% CO2 for 48 h. Subsequently, the cells were lysed with a lysis buffer (Promega), and lysate was measured by detecting the relative luminescence unit (RLU) in the photometer (Promega) to measure luciferase activity^3^.

**Protein expression and purification**

The expression and purification of the RBD protein was performed as described previously^4^. Briefly, D614G_RBD, B.1.351_RBD, B.1.617.2_RBD and B.1.1.529_RBD were expressed and purified from 293F cells. In short, the DNA sequence was 6 x His labeled and D614G_RBD, B.1.351_RBD, B.1.617.2_RBD and B.1.1.529_RBD were cloned into pcDNA3.1 vector. The constructs were transfected into 293F cells. After 5 days, the supernatant was collected and centrifuged to discard cell debris. The cleared supernatant was passed through Ni-NTA agarose to enrich the His-labeled target protein and then eluted with Tris buffer containing imidazole. The purified protein was concentrated and the buffer was replaced with regular Tris buffer. The protein concentration was determined by the BCA assay method. Coomassie Blue was performed to confirm its purity.

**Surface plasmon resonance (SPR)**

The recombinant hACE2 protein was immobilized on a CM5 Sensor Chip (carboxymethylated dextran covalently attached to a gold surface) with the amine coupling kit (GE Healthcare). D614G_RBD, B.1.351_RBD, B.1.617.2_RBD, and B.1.1.529_RBD were pre-incubated with serial concentrations, including 4 nM, 2 nM, 1 nM, 0.5 nM, and 0.25 nM in the HEPS buffer and then injected (30 µl/min), respectively. The signals were recorded by the Biacore T100 instrument with the standard protocols.

**Statistical analysis**

The statistical details of the specific experiment, including the statistical test used, number of samples, mean values, standard error of the mean (SD) and p values, were described in the figure legends. For comparison between each group with the mean of every other group within a dataset containing more than two groups, one-way ANOVA with Tukey’s multiple comparisons test was used. **p* ≤ 0.05, ***p* ≤ 0.01, ****p* ≤ 0.001, *****p* ≤ 0.0001. Statistical analyses were conducted utilizing Graphpad Prism 8.0 or Microsoft Excel.

References

1 Liu, B. *et al.* Recovered COVID-19 patients with recurrent viral RNA exhibit lower levels of anti-RBD antibodies. *Cellular & Molecular Immunology* **17**, 1098-1100, doi:10.1038/s41423-020-00528-0 (2020).

2 Pan, T. *et al.* Significantly reduced abilities to cross-neutralize SARS-CoV-2 variants by sera from convalescent COVID-19 patients infected by Delta or early strains. *Cell Mol Immunol* **18**, 2560-2562, doi:10.1038/s41423-021-00776-8 (2021).

3 Korber, B. *et al.* Tracking Changes in SARS-CoV-2 Spike: Evidence that D614G Increases Infectivity of the COVID-19 Virus. *Cell* **182**, 812-827 e819, doi:10.1016/j.cell.2020.06.043 (2020).

4 Ma, X. *et al.* Nanoparticle Vaccines Based on the Receptor Binding Domain (RBD) and Heptad Repeat (HR) of SARS-CoV-2 Elicit Robust Protective Immune Responses. *Immunity*, doi:10.1016/j.immuni.2020.11.015 (2020).
